# Kinetics of Glycoxidation of Bovine Serum Albumin by Methylglyoxal and Glyoxal and its Prevention by Various Compounds

**DOI:** 10.3390/molecules19044880

**Published:** 2014-04-17

**Authors:** Izabela Sadowska-Bartosz, Sabina Galiniak, Grzegorz Bartosz

**Affiliations:** 1 Department of Biochemistry and Cell Biology, University of Rzeszów, Zelwerowicza St. 4, PL 35-601 Rzeszów, Poland; 2 Department of Molecular Biophysics, University of Łódź, Pomorska 141/143, 90-236 Łódź, Poland

**Keywords:** glycation, kinetics, methylglyoxal, glyoxal, antioxidants

## Abstract

The aim of this study was to compare several methods for measurement of bovine serum albumin (BSA) modification by glycoxidation with reactive dicarbonyl compounds (methylglyoxal ‒ MGO and glyoxal ‒ GO), for studies of the kinetics of this process and to compare the effects of 19 selected compounds on BSA glycation by the aldehydes. The results confirm the higher reactivity of MGO with respect to GO and point to the usefulness of AGE, dityrosine and *N*′-formylkynurenine fluorescence for monitoring glycation and evaluation of protection against glycation. Different extent of protection against glycation induced by MGO and GO was found for many compounds, probably reflecting effects on various stages of the glycation process. Polyphenols (genistein, naringin and ellagic acid) were found to protect against aldehyde-induced glycation; 1-cyano-4-hydroxycinnamic acid was also an effective protector.

## 1. Introduction

One of undesired and unavoidable consequences of metabolism are non-enzymatic reactions of aldehyde/ketone groups of reducing sugars with other cellular components, mainly proteins (glycation or Maillard reaction). Glycation can also occur with highly reactive sugar derivatives such as α-oxoaldehydes, glyoxal (GO), methylglyoxal (MGO) and 3-deoxyglucosone. α-Oxoaldehydes, which are important precursors of advanced glycation end products (AGEs), can be formed endogenously by degradation of glucose and early glycation products. GO is formed by lipid peroxidation and the degradation of monosaccharides, saccharide derivatives and glycated proteins. MGO can be generated under physiological conditions by non-enzymatic elimination of phosphate from dihydroacetone phosphate or glyceraldehyde-3-phosphate [1]. Additionally, MGO can also be formed as a side-product of different metabolic pathways (e.g., by enzymatic elimination of phosphate from glycerone phosphate, glyceraldehyde 3-phosphate or from 3-aminoacetone in the threonine catabolism, during lipid peroxidation and from the degradation of DNA) [2]. MGO is produced in a high amount in diabetes mellitus enzymatically e.g. by MGO synthase and cytochrome P450 IIE1 and also by non-enzymatic reactions because of the increase in non-enzymatic glycation [3]. In an advanced stage of diabetic complications in the case of ischemic tissue damage, hypoxia itself enhances local production of MGO in the ischemic zone [4]. Blood plasma MGO concentration was estimated to be in the order of 1–10 μM range [3]. Recent estimates of the concentrations of MGO and GO in human blood plasma are in the range 70–120 nM, while cellular concentrations of MGO are 1–5 μM and those of GO are 0.1–1 μM [5]. Increased MGO levels were found in whole blood samples of non-insulin-dependent diabetic patients (mean: 286.8, range: 54.7–2370 pmol/g blood) when compared to control subjects (mean: 79.8, range: 25.3–892.9 pmol/g blood) [4].

MGO in the free form is very unstable, has short biological survival times, and sophisticated techniques are required for its quantitative evaluation [6]. It has been reported that MGO binds to and modifies a number of proteins [7], and damages DNA, thus causing cellular toxicity [8]. Modification of human serum albumin (HSA) by MGO produced hotspot modification of Arg-410 with hydroimidazolone, *N*^δ^-(5-hydro-5-methyl-4-imidazolon-2-yl)-ornithine (MG-H1) residue formation. Modification of Arg-410 by MGO was found in albumin glycated *in vivo*. MG-H1 residue formation in albumin and other plasma proteins is increased in clinical diabetes and end stage renal disease [5]. Molecular dynamics and modeling studies indicate that hydroimidazolone formation causes structural distortion leading to disruption of arginine-directed hydrogen bonding and loss of electrostatic interaction. Hydroimidazolone formation at Arg-410 inhibits drug binding and esterase activity [9]. A specific GO-derived AGE is a lysine-lysine crosslinking structure named GO-lysine dimer or GOLD. Investigators have reported an age-related increase of GOLD in human lens proteins as well as in diabetes [1]. The posttranslational modification of proteins by MGO is believed to contribute to aging, as well as to development of a number of diseases, including cancer, diabetes and other disorders. MGO-induced AGEs on mitochondrial proteins leads to a decline of mitochondrial function and have been hypothesized to act as a contributing factor in the phenomenon of so-called “metabolic memory” [10].

Glycation is usually catalyzed by metals and associated with generation of reactive oxygen species (ROS) and oxidation so it is often referred to as glycoxidation although reducing derivatives are also formed in the course of this process [11]. Proteins, because of their high abundance, are the main substrate for glycoxidation. Glycoxidation is initiated by formation of early glycation products such as Schiff bases and Amadori products. Further modifications of the early stage glycation products, such as rearrangement, oxidation, polymerization and cleavage give rise to irreversible conjugates (AGEs). Although AGE formation takes place under physiological conditions, it is accelerated in hyperglycemia. Accumulation of glycation products is observed in human and animal tissues during aging and is associated with various diseases including, first of all, diabetes and diabetic nephropathy, microangiopathy and atherosclerosis [12,13,14].

The serum levels of AGEs have been found to be elevated also in other diseases, including, *i.a.* multiple sclerosis [15] and amyotrophic lateral sclerosis [16], and correlated with the severity of cognitive impairment in patients with cerebrovascular disease [13]. Studies of proteins containing either early stage glycation products or AGEs have become of great interest due to the suspected effects of glycation on protein function and tissue damage in diabetes. This effect has been well-studied for proteins with long life spans, such as collagen and lens crystallin, as well for some proteins with shorter life spans, such as hemoglobin. However, there has also been an increasing interest in the glycation of HSA and closely-related proteins (e.g., bovine serum albumin, BSA) [17].

Albumin represents the most abundant protein, constituting some 50% of the protein present in the plasma of normal healthy individuals and has a wide variety of physiological and pharmacological functions including the maintenance of oncotic pressure, binding and transport of diverse small size metabolites such as metal ions, fatty acids, bilirubin, drugs and nitric oxide, and contribution to the antioxidant capacity of blood plasma [2]. *In vivo*, the proportion of glycated albumin in healthy persons is between 1% and 10%; this proportion may increase two- to threefold in diabetes mellitus [18]. Therefore, this protein is not only a convenient but also very relevant model for studies of the reactions of glycoxidation.

In view of the growing role of AGEs in diabetes and age associated pathologies, it has been suggested that inhibition of the formation of AGEs may prevent the progression of diabetic complications and slow down the ageing process. Pharmacological agents, such as aminoguanidine, tenilsetam, carnosine, metformin and pyridoxamine, have been investigated for inhibiting the formation of AGEs and/or the development of diabetic complications. Nevertheless, all of these compounds have side effects. It is thus critical to develop effective and safe agents to protect diabetics from complications [8]. Therefore, on-going screening and development of novel compounds that offer combined antioxidant and antiglycation properties will benefit to the treatment of diabetes mellitus [19].

The aim of the present study was twofold. Firstly, we wanted to compare several methods for measurement of BSA (as a model protein) modification by reactive dicarbonyl compounds (GO and MGO) for monitoring the kinetics of this process. Secondly, we compared the effects of 19 selected compounds on BSA glycoxidation induced by reactive dicarbonyls in search of compounds which can efficiently prevent this process. The compounds chosen included natural polyphenols (ellagic acid, genistein, naringin), natural (ascorbic acid, lipoic acid, cysteamine, Na-pyruvate, *para*-aminobenzoic acid) and synthetic (captopril, tiron, TEMPO, 4-hydroxy-TEMPO, 2,2,6,6-tetramethyl-4-(nonanoyl-amino)piperidin-1-yl]oxyl) antioxidants, an inhibitors of glycolysis (3**-**bromopyruvic acid), an antidiabetic drug (metformin), and a cyclic polyol (quinic acid). We included also 1-cyano-4-hydroxy-cinnamic acid, a compound we found to inhibit glycation by reducing sugars (submitted) and its parent compound, 4-hydroxycinnamic acid, and environmental contaminant bisphenol A, known to affect glucose homeostasis and be a factor in the etiopathogenesis of diabetes [20].

## 2. Results

**Figure 1:**
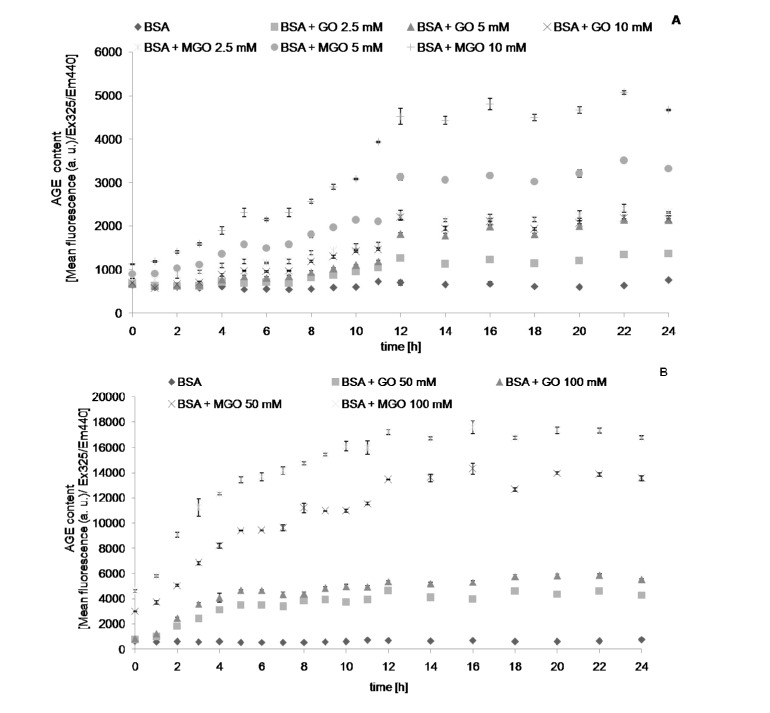
The time course of AGE fluorescence during incubation of BSA (0.09 mM) with 2.5, 5 and 10 mM (**A**), and 50 and 100 mM (**B**) GO and MGO at 37 °C.

**Figure 2:**
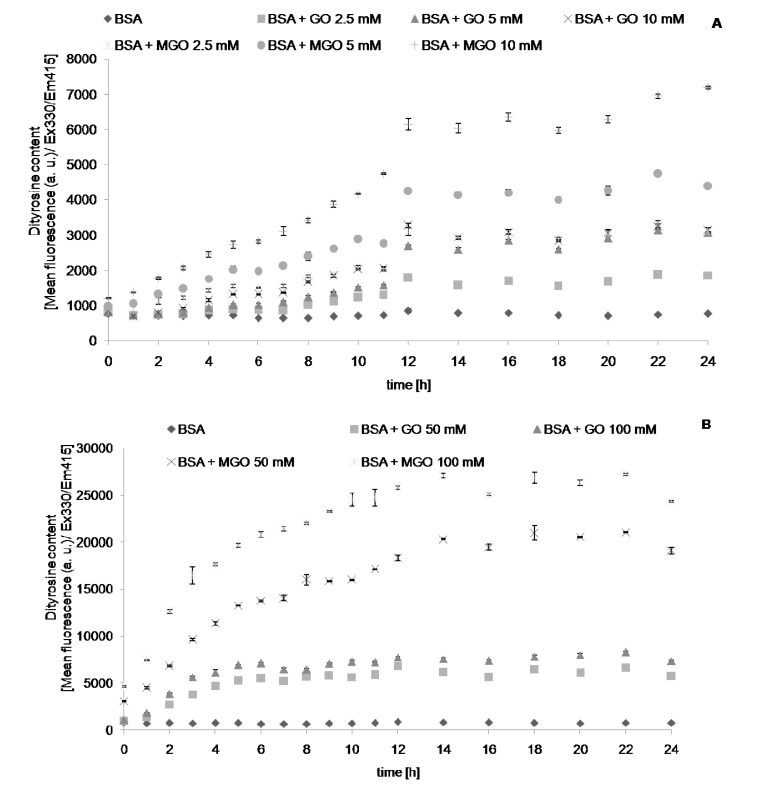
The time course of dityrosine fluorescence during incubation of BSA (0.09 mM) with 2.5, 5 and 10 mM (**A**), and 50 and 100 mM (**B**) GO and MGO at 37 °C.

**Figure 3:**
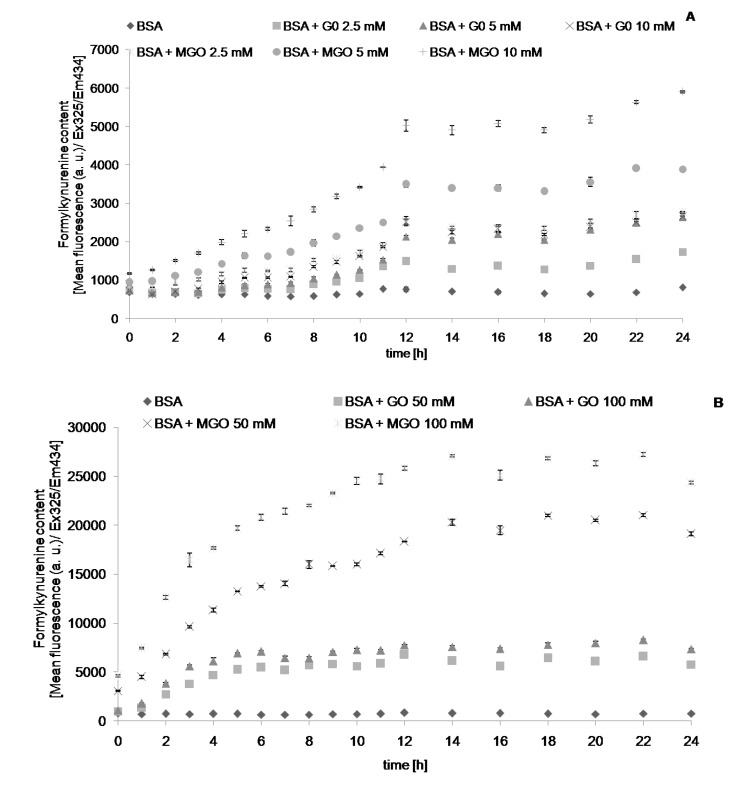
The time course of *N*′-formylkynurenine fluorescence during incubation of BSA (0.09 mM) with 2.5, 5 and 10 mM (**A**), and 50 and 100 mM (**B**) GO and MGO at 37 °C.

**Figure 4:**
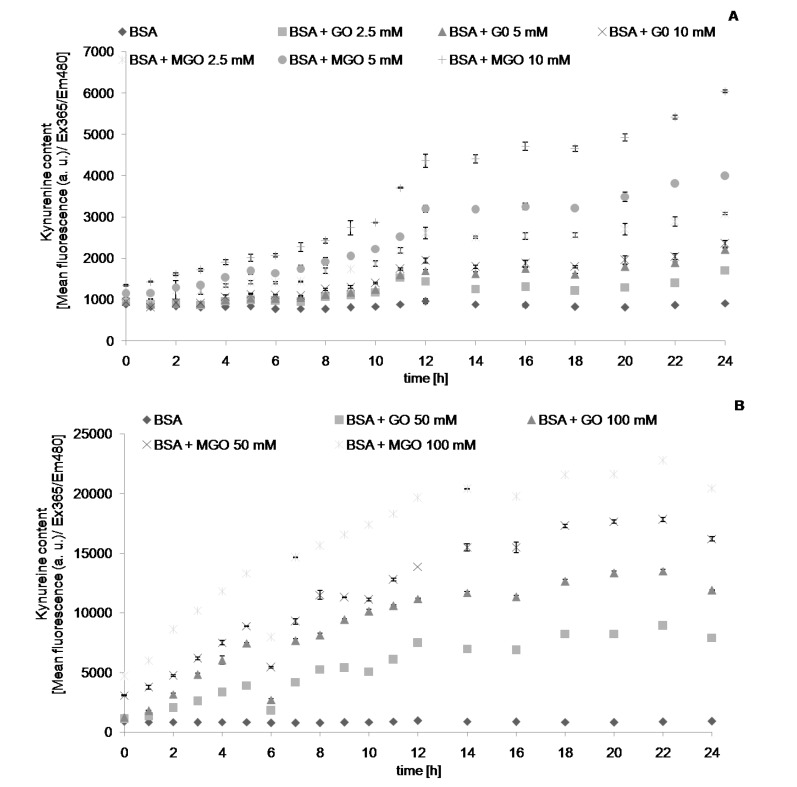
The time course of kynurenine fluorescence during incubation of BSA (0.09 mM) with 2.5, 5 and 10 mM (**A**), and 50 and 100 mM (**B**) GO and MGO at 37 °C.

**Figure 5:**
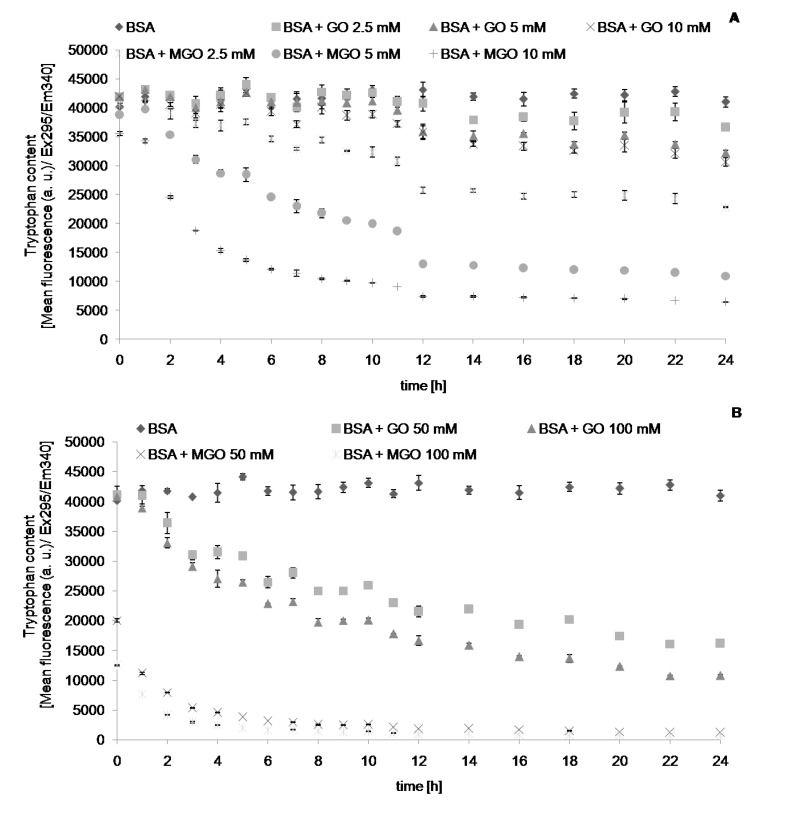
The time course of tryptophan fluorescence during incubation of BSA (0.09 mM) with 2.5, 5 and 10 mM (**A**), and 50 and 100 mM (**B**) GO and MGO at 37 °C.

We followed the changes in the values of chosen parameters describing protein glycation and oxidative modifications during and 24-h incubation with reactive aldehydes, MGO and GO. The level of AGEs, determined fluorimetrically, increased with time during incubation with the aldehydes, until reaching a plateau level. Noteworthy, this parameter increased also (albeit at a low rate) in control samples containing only albumin. This casts doubt on the specificity of this parameter as an index of AGE formation. Apparently, the fluorimetric measurement is not fully specific, most probably being contributed by other protein oxidation products. The time-course of formation of AGEs (Figure 1), dityrosine (Figure 2), *N*′-formylkynurenine (Figure 3) and kynurenine (Figure 4) showed a similar behavior. The level of tryptophan fluorescence decreased progressively until a plateau (Figure 5), reflecting a glycoxidation-induced destruction of these residues and/or changes in protein conformation resulting in exposure of tryptophan residue to a more polar environment where the yield of fluorescence is lower [21,22]. AOPP and carbonyl levels increased progressively during incubation.

**Figure 6:**
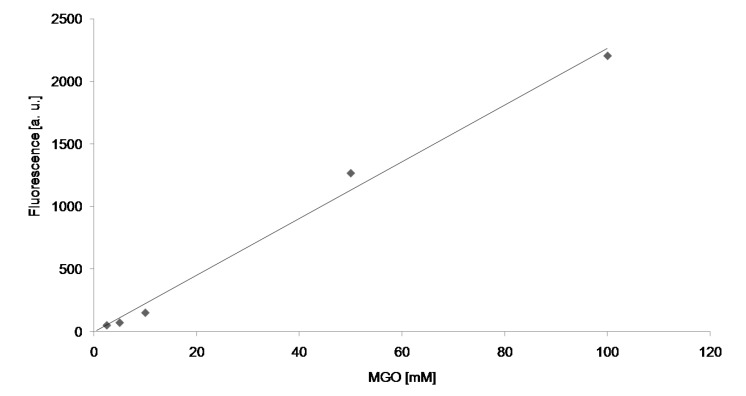
Example of plots of dependence of the rate of AGE formation on the concentration of MGO.

In order to get some quantitative information on the rate of protein modifications by glycoxidation, it was assumed that the initial course of the reactions is linear (up to at least 3 h) and the initial reaction rates estimated from kinetic curves were plotted *vs.* concentration of the aldehydes. Such plots were proportional (Figure 6) demonstrating that reactions were first order with respect to aldehydes. From the slopes of the plots, apparent second-order rate constants of the reactions were calculated (Table 1).

**Table 1:** Apparent second-order rate constants for the reactions of glycoxidative modifications of BSA by glyoxal and methylglyoxal (in fluorescence units^−1^ × mM^−1^ × h).

Modification	Glyoxal	Methylglyoxal
AGE formation	10.4	22.7
Dityrosine formation	48.9	121.3
*N*-Formylkynurenine formation	11.3	27.1
Kynurenine formation	12.5	19.0

In search for compounds which could effectively inhibit glycation, we have compared the effects of 19 compounds on the glycation induced by both aldehydes. Results of this screening are shown in Table 2 and Table 3. We excluded tryptophan fluorescence from these experiments due to interference with several compounds.

The effects of the compounds studied on MGO- and GO-induced glycation of BSA were different for both aldehydes and were generally similar for AGE, dityrosine and N’-formylkynurenine formation while their effect on the kynurenine fluorescence was often different, most likely due to interference with glycation products or additives.

3-Bromopuryvate enhanced protein modifications by GO and MGO as indicated by all the parameters shown. A similar effect was observed for bisphenol A (except for MGO-induced kynurenine formation). Ascorbic acid strongly enhanced BSA modification by GO), the effect being similar but much lower for MGO. Metformin potentiated BSA glycation by both aldehydes for most parameters measured. Captopril enhanced protein modification by GO while showing a small inhibitory effect in the case of MGO. Cysteamine inhibited formation of all derivatives studied by MGO while having no effect on GO-induced modifications, except for enhancement of kynurenine formation by GO. Tiron and TEMPO had no significant effect on AGE, dityrosine and *N*′-formylkynurenine formation induced by GO but inhibited significantly formation of these derivatives by MGO, while enhancing kynurenine formation by both compounds. Another nitroxide studied, 2,2,6,6-tetramethyl-4-[(nonanoylamino)piperidin-1-yl]oxyl, did not affect AGE, dityrosine and *N*′-formylkynurenine formation and enhanced kynurenine formation by MGO, while inhibiting generation of all derivatives studied by GO. Quinic acid and *para*-aminobenzoic acid enhanced the formation of all derivatives studied by GO and decreased significantly their formation by MGO (except for kynurenine which was enhanced). Pyruvate enhanced formation of all derivatives studied by GO, had no effect on the formation of AGE, dityrosine and *N*′-formylkynurenine, and decreased kynurenine formation by MGO. Lipoic acid had no significant effect on AGE, dityrosine and *N*′-formylkynurenine formation and decreased formation of these derivatives by MGO while enhancing kynurenine formation by both aldehydes. Naringin, genistein and ellagic acid were inhibitory except for enhancing kynurenine formation induced by GO. 1-Cyano-4-hydroxycinnamic acid was inhibitory in all cases. Hydroxycinnamic acid was slightly inhibitory in most cases for both aldehydes.

**Table 2:** Inhibition by various compounds of AGE, dityrosine, *N*′-formylkynurenine and kynurenine in BSA induced by methylglyoxal (MGO). Values expressed as % of fluorescence increase in samples incubated without any additives (with DMSO only for compounds introduced as solutions in DMSO), given as mean values ± SD from 6 independent experiments. * *p* < 0.05 or less.

Compound	AGE	Dityrosine	*N*′-formylkynurenine	Kynurenine
2,2,6,6-Tetramethyl-4-[(nonanoylamino)piperidin-1-yl]oxyl	97.8 ± 4.78	96.25 ± 4.4	97.55 ± 4.41	326.09 ± 11.77 *
3-Bromopyruvate	216.01 ± 7.89 *	208.88 ± 6.92 *	221.49 ± 7.92 *	120.5 ± 8.34 *
4-Hydroxy-TEMPO	80.54 ± 5.79 *	75.62 ± 4.61 *	78.33 ± 5.27 *	162.6 ± 6.02 *
Ascorbic acid	104.4 ± 2.09 *	111.97 ± 2.85 *	109.21 ± 2.49 *	182.54 ± 5.77 *
Bisphenol A	155.2 ± 5.22 *	155.17 ± 3.91 *	154.4 ± 3.68 *	92.07 ± 4.51 *
Captopril	88.84 ± 3.17 *	93.46 ± 3.4 *	93.56 ± 4.41 *	13.78 ± 0.45 *
1-Cyano-4-hydroxycinnamic acid	3.5 ± 0.213 *	3.94 ± 0.09 *	3.69 ± 0.16 *	81.72 ± 5.24 *
Cysteamine	65.19 ± 2.69 *	63.73 ± 2.84 *	64.36 ± 2.97 *	30.95 ± 0.47 *
Ellagic acid	47.02± 0.51 *	35.49 ± 0.22 *	45.6 ± 0.33 *	48.1 ± 1.32 *
Genistein	27.17 ± 0.31 *	25.98 ± 0.34 *	27.92 ± 0.52 *	85.90 ± 3.79 *
4-Hydroxycinnamic acid	91.78 ± 3.39 *	92.35 ± 2.54 *	89.67± 3.67 *	89.4 ± 3.35 *
Lipoic acid	79.19 ± 2.42 *	80.86 ± 2.33 *	81.69 ± 2.79 *	123.25 ± 4.41 *
Metformin	178.72 ± 5.34 *	100.93 ± 2.6	163.44 ± 4.47 *	146.51 ± 3.50 *
Na-pyruvate	100.52 ± 1.9	95.09 ± 1.75 *	101.61 ± 2.22	63.33 ± 1.84
Naringin	19.84 ± 0.68	16.87 ± 0.26	18.79 ± 0.49	69.32 ± 2.24 *
*para*-Aminobenzoic acid	46.25 ± 1.28 *	46.43 ± 1.35 *	46.59 ± 1.21 *	111.84 ± 6.47 *
Quinic acid	57.29 ± 3.45 *	57.27 ± 3.42 *	61.35 ± 3.96 *	128.99 ± 5.57 *
TEMPO	78.11 ± 3.45 *	75.09 ± 3.22 *	80.35 ± 3.48 *	106.35 ± 4.13 *
Tiron	68.16 ± 4.16 *	75.4 ± 4.15 *	70.53 ± 4.54 *	326.09 ± 11.77 *

**Table 3:** Inhibition by various compounds of AGE, dityrosine, N’-formylkynurenine and kynurenine in BSA induced by glyoxal (GO). Values expressed as % of fluorescence increase in samples incubated without any additives (with DMSO only for compounds introduced as solutions in DMSO), given as mean values ± SD from 6 independent experiments.* *p* < 0.05 or less.

Compound	AGE	Dityrosine	*N*′-formylkynurenine	Kynurenine
2,2,6,6-Tetramethyl-4-[(nonanoylamino)piperidin-1-yl]oxyl	80.94 ± 10.25 *	82.6 ± 7.46 *	82.03 ± 8.21 *	74.68 ± 18.28
3-Bromopyruvate	228.89 ± 11.14 *	201.68 ± 8.82 *	225.71 ± 9.33 *	544.55 ± 28.26 *
4-Hydroxy-TEMPO	106.79 ± 11.96	104.33 ± 10.21	109.75 ± 11.15	116.48 ± 19.23
Ascorbic acid	217.49 ± 3.3 *	226.31 ± 3.09 *	214.49 ± 2.97 *	1233.03 ± 5.13 *
Bisphenol A	211.51 ± 4.41 *	173.97 ± 5.28 *	201.74 ± 3.91 *	645.93 ± 17.76 *
Captopril	118.21 ± 5.33 *	114.82 ± 5.69 *	120.55 ± 5.96 *	160.94 ± 11.56 *
1-Cyano-4-hydroxycinnamic acid	2.48 ± 0.45 *	3.60 ± 0.36 *	3.00 ± 0.45 *	0.78 ± 1.27 *
Cysteamine	97.86 ± 14.26	90.96 ± 9.168	96.79 ± 10.56	221.00 ± 23.69 *
Ellagic acid	50.67 ± 0.53 *	31.76 ± 0.17 *	46.59 ± 0.45 *	137.45 ± 2.28 *
Genistein	48.07 ± 1.11 *	42.62 ± 1.01 *	48.5± 0.99 *	121.83 ± 7.36 *
4-Hydroxycinnamic acid	85.28 ± 6.09 *	89.68 ± 8.33 *	90.86 ± 11.44	117.76 ± 46.25
Lipoic acid	98.7 ± 4.86	97.7 ± 4.13	105.12 ± 4.19 *	122.04 ± 8.49 *
Metformin	112.98 ± 7.01 *	118.51 ± 5.75 *	118.95 ± 6.17 *	140.2 ± 11.96 *
Na-pyruvate	146.55 ± 6.16 *	138.77 ± 11.67 *	143.83 ± 11.52 *	221.12 ± 22.40 *
Naringin	37.86 ± 0.59 *	28.18 ± 0.57 *	35.88 ± 0.22 *	206.21± 2.45 *
*para*-Aminobenzoic acid	94.7 ± 4.86	96.59 ± 4.42	99.59 ± 4.22	129.67± 8.2 *
Quinic acid	147.82 ± 10.8 *	145.38 ± 8.21 *	149.06 ± 8.75 *	213.44 ± 14.32 *
TEMPO	101.77 ± 6.94	98.71 ± 6.32	113.51± 10.75 *	133.52 ± 17.71 *
Tiron	95.96 ± 5.37	113.84 ± 9.77 *	106.34 ± 10.88	170.80 ± 9.68 *

## 3. Discussion

We studied the reaction of reactive aldehydes, GO and MGO, with proteins using a model relevant protein, BSA, a 66.7-kDa protein rich in lysine (59; 10.1%) and arginine residues (23; 3.9%). Non-enzymatic glycosylation of albumin *in vivo* occurs at multiple sites corresponding to arginine, lysine and cysteine residues. These structural modifications of albumin induced by glycation include an increase in molecular weight and higher exposure of hydrophobic sites to the solvent. By contrast, aggregate formation, which is induced by glycation, is not necessarily associated with secondary structure modification. Albumin is the main plasma protein; alteration of its properties and physiological functions may be thus of physiological significance [2]. The level of glycated HSA is used as a short-to-intermediate term marker for glycemic control in diabetes [17].

We monitored fluorimetrically the formation of AGEs, which is an established marker of glycation. Simultaneously, other fluorimetric parameters and AOPP formation was followed. Changes in all parameters studied occurred in parallel, confirming validity of the concept of glycoxidation with respect to the system studied. Our results demonstrate that simple fluorimetric tests of protein glycoxidative modifications reflecting damage to tryptophan and tyrosine residues, viz. dityrosine and *N*′-formylkynurenine formation, and loss of tryptophan fluorescence, are convenient measures of the progress of protein glycation and protection against glycation by various compounds, apart from the formation of AGEs. Tryptophan fluorescence and kynurenine fluorescence do not seem to be appropriate markers in studies of protective effects of various substances, perhaps being more subject to interference. We studied also oxidation of thiol groups, which followed the pattern of other changes measured but the extent of changes makes this parameter less convenient as a marker of glycoxidation (not shown).

Reactive dicarbonyls are expected to contribute to the glycation of albumin *in vivo*. Results of this study confirm the higher reactivity of MGO with respect to GO, which suggests that *in vivo* MGO rather than GO is the main dicarbonyl compound responsible for albumin glycation.

On the basis of kinetic studies, optimal time for studies of the effect of additives on the course of protein modification was chosen. This time (12 h) corresponds to the end of the period of time-dependent increase in glycation before reaching a plateau. Often, prolonged incubation times are used for checking the effects of various additives on glycation, apparently corresponding to the plateau phase. Such an approach can hide effects on the rate of glycation due to the catch up effect in the presence of inhibitors, though may cover the action of additives on the stability/decomposition of the glycation products. The time of incubation may be one reason for discrepancy of literature results concerning the effects of additives on the extent of glycation. The results of the present study report mainly effects of various substances on the rate of glycation.

In search of compounds which modify the glycation by reactive carbonyls, we studied the effects of various compounds on the BSA modification by MGO and GO. If glycoxidation involves oxidation reactions, antioxidant compounds should be expected to inhibit this process. However, both thiols used, cysteamine and captopril, inhibited the modification induced by MGO only, while having no effect on those induced by GO and enhancing GO-induced kynurenine formation; captopril enhanced BSA modification by GO. Captopril was previously reported to inhibit glycation of albumin and IgG by glucose [23,24]. Ascorbic acid, an antioxidant, can have prooxidant properties especially in various *in vitro* systems in the presence of metal ions, hard to avoid as contaminants. This compound has been reported both to induce and inhibit glycation in various systems [25,26,27]. In our system, ascorbic acid promoted glycation by both aldehydes, as evidenced by all parameters measured. Lipoic acid showed moderate protective affects against MGO-induced glycation, having no effect on GO-induced glycation and enhancing kynurenine formation. Lipoic acid has been reported to inhibit glycation *in vivo* [28]; however, this effect is based on the metabolic reduction of this compound which cannot take place in our simple *in vitro* system. Pyruvate is an antioxidant, reacting with reactive oxygen species, especially hydrogen peroxide [29] and was reported to inhibit sugar-induced glycation *in vitro* and *in vivo* [30,31]. However, in our system pyruvate was ineffective as inhibitor of MGO-induced glycation (except for decreasing kynurenine fluorescence) and enhanced GO-induced glycation. This result may suggest a lack of significant involvement of hydrogen peroxide in the glycation induced by reactive aldehydes. *para*-Aminobenzoic acid is an intermediate in the bacterial synthesis of folate and an antioxidant [32]. In our study, it inhibited BSA glycation induced by MGO and had no effect on GO-induced glycation (except for increasing kynurenine fluorescence for both aldehydes).

Tiron is an antioxidant that reacts with superoxide at a high rate, and a metal chelator [33,34], so it can be expected to inhibit protein glycation. This compound decreased significantly the level of BSA modification by MGO but had no effect on GO-induced glycation, enhancing the kynurenine fluorescence in both cases.

Nitroxides are effective antioxidants of pseudo-superoxide dismutase activity, preventing Fenton reaction by oxidizing transition metal ions [35] and can be also expected to inhibit glycoxidation. We used three nitroxides: Tempo, 4-hydroxy-Tempo (Tempol) and 2,2,6,6-tetramethyl-4-[(nonanoylamino)piperidin-1-yl]oxyl as potential glycation-interfering agents. The latter compound seemed more promising due to the presence of an amino group. Somewhat surprisingly, Tempo inhibited MGO-induced BSA glycation not affecting GO-induced glycation (enhancing kynurenine fluorescence in both cases), 4-hydroxy-Tempo was ineffective while 2,2,6,6-tetramethyl-4-[(nonanoylamino)piperidin-1-yl]oxyl had no effect on MGO-induced glycation (enhancing kynurenine fluorescence) but inhibited GO-induced glycation.

Polyphenols, especially flavonoids, have been demonstrated to be effective inhibitors of glycation. We found significant inhibition of MGO- and GO-induced BSA glycation by naringin, genistein and ellagic acid (except for enhancement of kynurenine fluorescence induced by GO). Previously, inhibition of glycation has been demonstrated for other polyphenols, including quercetin, genistein, tannic acid and gallic acid [7,8,36,37,38]. Plant-derived polyphenols appear thus to be promising anti-glycating agents although their poor bioavailability may pose practical problems. 4-Hydroxycinnamic acid is also a compound of antioxidant properties [39]. This compound showed some inhibiting properties against MGO- and GO-induced glycation.

Quinic acid, a cyclitol, was reported to inhibit glycation induced by glucose plus fructose [40]. In our system, it inhibited MGO-mediated glycation (though enhancing kynurenine fluorescence) while enhancing GO-induced glycation.

Metformin is a well-known inhibitor of the effects of glycation *in vivo*, partly due to inhibition of expression of inhibitors for RAGE [41], but was also reported to inhibit glycation *in vivo* and *in vitro* [42,43]. Interestingly, this compound enhanced BSA glycation by MGO and GO, which indicates that the effects of this compound *in vivo* are due mainly to other effects than inhibiting protein modification by reactive aldehydes.

3-Bromopyruvate is a reactive derivative of pyruvate, inhibitor of glycolysis and a promising anticancer drug [44]. This compound enhanced glycation significantly, which may contribute to the side-effects of anticancer therapy by 3-bromopyruvate.

Bisphenol A is one of the most common chemicals to which we are exposed in everyday life. It is the building block of polycarbonate plastic and is also used in the manufacture of epoxy resins found in many common consumer products. Bisphenol A enhanced glycation by both MGO and GO, which may be a new facet of the physiological effects of this environmental agent reported to elevate the risk of diabetes [20].

1-Cyano-4-hydroxycinnamic acid is known mainly as an inhibitor of monocarboxylate transporters [45]. This compound was a strong inhibitor of glycation by both MGO and GO, which may points to a new class of potential inhibitors of glycation. 4-Hydroxycinnamic acid is also a compound of antioxidant properties [39]. This compound showed some inhibiting properties against MGO- and GO-induced glycation but its effect were much smaller with respect to its cyano derivative. The mechanism of anti-glycating action of 1-cyano-4-hydroxycinnamic acid is unknown at present; it can be hypothesized that it is due to trapping MGO/GO, as it was reported for genistein [8].

## 4. Experimental

All basic reagents were from Sigma-Aldrich (Poznan, Poland) unless indicated otherwise. Glyoxal and methylglyoxal were used within a month after delivery and their working solutions were prepared immediately before use. Genistein, 4-hydroxycinnamic acid, naringin and quinic acid were purchased from Santa Cruz Biotechnology, Inc. (Dallas, TX, USA). 2,2,6,6-Tetramethyl-4-(nonanoyl-amino)piperidin-1-yl]oxyl was synthesized by Dr. Janusz Skolimowski (Department of Organic Chemistry, University of Łódź, Poland). Bovine serum albumin (BSA, 96% purity) was dissolved in 0.1 M sodium phosphate buffer, pH 7.4 at a concentration of 0.1 mM. As AGE-inducing agents, two dicarbonylic compounds (GO and MGO) were applied. The incubation mixtures contained BSA at a final concentrations of 90 μM and 2.5, 5, 10, 50 and 100 mM GO or MGO. The concentration range (2.5–100 mM) was chosen on the basis of similar publications [7,8,46,47,48,49,50]. The lowest concentration of aldehydes was determined on the basis of studies on cell cultures in which 1 h treatment with MGO concentrations up to 2.5 mM did not appear to have detectable deleterious effects on the viability of human leukemia 60 cells [51].

These concentrations are well above physiological levels but are convenient for modeling in a relatively short time the processes occurring physiologically over weeks or months. For measurements of the effects of other compounds on the process of glycation, BSA (90 μM) was incubated with GO or MGO (2.5 mM) for 12 h. The compounds studied as modifiers of glycation were added at a final concentration of 1 mM. This concentration was selected on the basis of other *in vitro* studies, in proportion to the high concentrations of the glycating agents [8,52].

The samples were incubated in closed vials at the temperature of 37 °C for 24 h. Selected parameters were measured in triplicate by applying 200 µL of the sample on the plate every hour. Fluorescence was measured at a wavelength of 325/440 nm (AGE), 330/415 nm (dityrosine), 325/434 nm (*N*′-formylkynurenine) and 365/480 nm (kynurenine) [15].

Advanced oxidation protein products (AOPP) concentration was estimated according to Witko-Sarsat *et al.* [53] using 200 µL of the sample and adding 10 µL of 1.16 M sodium iodide and 20 µL of acetic acid. The absorbance was measured immediately at a wavelength of 340 nm. AOPP concentration was expressed in nmol/mg protein.

Fluorimetric and absorptiometric measurements were done in an Infinite 200 PRO Multimode Reader (Tecan Group Ltd., Männedorf, Switzerland).

Data were given in the form of arithmetic mean values and standard deviations. Differences between means were analyzed using two-tailed Student’s “t” test, according to the formula t_d_ = (׀x - x_o_׀/s) × 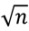, where x- mean value of the sample with additive expressed in %, x_o_: value for control sample assumed as 100%, s: standard deviation, n: number of experiments (usually n = 3). The calculations were made in Excel.

## 5. Conclusions

In summary, this study points to the usefulness of simple fluorimetric parameters of protein glycooxidative damage for monitoring the kinetics of and protection against glycoxidation. The study demonstrated the possibility of inhibition of MGO- and GO-induced glycation by antioxidants and some other compounds documenting differences in the protection both between bifunctional aldehydes and reducing sugars (by comparison with literature data) and between the two aldehydes applied. The reason for the latter phenomenon is not easy to explain but may be perhaps due to the difference in the rate of glycoxidation. Measurements of more rapidly proceeding MGO-induced glycation may reflect changes at later stages of this process, not occurring yet in GO-induced glycation, and subject to different types of interference. This question is currently under study.
